# Nitric-Oxide-Releasing Dexamethasone Derivative NCX-1005 Improves Lung Function and Attenuates Inflammation in Experimental Lavage-Induced ARDS

**DOI:** 10.3390/pharmaceutics13122092

**Published:** 2021-12-05

**Authors:** Petra Kosutova, Maros Kolomaznik, Andrea Calkovska, Daniela Mokra, Pavol Mikolka

**Affiliations:** 1 Biomedical Centre Martin, Jessenius Faculty of Medicine in Martin, Comenius University in Bratislava, 03601 Martin, Slovakia; petra.kosutova@uniba.sk (P.K.); maros.kolomaznik@uniba.sk (M.K.); 2 Department of Physiology, Jessenius Faculty of Medicine in Martin, Comenius University in Bratislava, 03601 Martin, Slovakia; andrea.calkovska@uniba.sk (A.C.); daniela.mokra@uniba.sk (D.M.)

**Keywords:** NCX-1005, nitro-steroid, nitric oxide donor, dexamethasone, ARDS, animal model, lung function, inflammation, oxidative stress

## Abstract

Acute respiratory distress syndrome (ARDS) is a common complication of critical illness and remains a major source of morbidity and mortality in the intensive care unit (ICU). ARDS is characterised by diffuse lung inflammation, epithelial and endothelial deterioration, alveolar–capillary leak and oedema formation, and worsening respiratory failure. The present study aimed to investigate the anti-inflammatory activity of nitric-oxide-releasing dexamethasone derivative NCX-1005 as a potential novel drug for ARDS. Adult rabbits with lavage-induced ARDS were treated with dexamethasone *i.v.* (0.5 mg/kg; DEX) and nitro-dexamethasone *i.v.* (0.5 mg/kg, NCX-1005) or were untreated (ARDS). Controls represented healthy ventilated animals. The animals were subsequently oxygen-ventilated for an additional 4 h and respiratory parameters were recorded. Lung oedema, inflammatory cell profile in blood and bronchoalveolar lavage, levels of the cytokines (IL-1β, IL-6, IL-8, TNF-α), and oxidative damage (TBARS, 3NT) in the plasma and lung were evaluated. Nitric oxide-releasing dexamethasone derivative NCX-1005 improved lung function, reduced levels of cytokines, oxidative modifications, and lung oedema formation to similar degrees as dexamethasone. Only NCX-1005 prevented the migration of neutrophils into the lungs compared to dexamethasone. In conclusion, the nitric oxide-releasing dexamethasone derivative NCX-1005 has the potential to be effective drug with anti-inflammatory effect in experimental ARDS.

## 1. Introduction

Acute respiratory distress syndrome (ARDS) is defined as an acute-onset syndrome, with bilateral diffuse infiltrates on chest radiography and non-cardiogenic respiratory failure [1]. ARDS is associated with diffuse alveolar damage and lung capillary endothelial injury leading to respiratory failure and oxygenation impairment. The early phase of ARDS is characterized by a neutrophil-mediated inflammation, and lung cell injury and apoptosis, with the subsequent influx of protein-rich fluid into the alveoli and oedema formation.

The changes in the microvascular endothelial structure and function play a central role in the acute inflammatory response. The endothelium becomes leaky and inflamed, allowing innate immune cells and humoral effector molecules to cross the barrier to the site of deterioration [2]. The mentioned overwhelming phenomena could lead to ARDS, initially with either direct (mainly pneumonia, aspiration of gastric contents) or indirect (mainly sepsis, multiple trauma) insults to the lung. Supportive therapy and mechanical ventilation are used for ARDS to prevent further ventilator-induced damage, reduce pulmonary oedema, and reduce hypoxaemia [3]. Currently, the management of ARDS is focused on minimizing iatrogenic damage, therapy of the primary cause inducing ARDS, and maintaining sufficient oxygenation, including lung-protective mechanical ventilation, fluid-restrictive management, and prone positioning [4,5].

However, it is essential to manage the systemic and pulmonary inflammatory response in ARDS. Despite the intense research in this field, no pharmacological therapy for ARDS has been shown to reduce short-term or long-term mortality. Glucocorticoids (GCs) have been considered as a potential therapy for ARDS due to their anti-inflammatory and immunomodulatory effects. GCs may improve oxygenation and airway pressures and may hasten radiographic improvement in patients with pneumonia, but concurrently, GCs are not associated with a consistent survival benefit and could be harmful if administration starts 14 or more days after ARDS diagnosis [6]. In addition, dexamethasone has shown a beneficial anti-inflammatory effect in different experimental models of ARDS (lavage or meconium induced) [7,8,9]. A previous study revealed that a low dose of dexamethasone (0.5 mg/kg b.w.) administered in the early phase of ARDS improved oxygenation and suppressed inflammation [9].

Despite the existing potent action characteristics and therapeutic efficiency of GCs, there is still a drive for the development of novel steroids. One way to do this is to synthesize GCs with different ratios of transrepression (mechanism of action through the inhibition of transcription factors, TFs) versus transactivation (mechanism of action through glucocorticoid response elements on DNA sequence, GREs) [10,11]. A different way to innovative GC development is to take advantage of nitric oxide (NO) anti-inflammatory actions. Many studies report that low concentrations of NO, especially if released slowly, produce marked anti-inflammatory effects. For instance, NO reduces leukocyte adhesion to microvascular endothelium [12] and extravasation [13], inhibits mast cell activation [14], and suppresses mediator synthesis (including its own) likely by an effect on TFs [15,16,17]. Derivatives of glucocorticoid releasing NO have shown an improved profile of pharmacological activity in terms of either enhanced anti-inflammatory efficacy or reduced side effects [18,19,20]. In lungs, the nitro-steroid was found to have an additional benefit over the parent molecule and enhanced bronchodilating action in vitro [21]. The design and study of NO-donating drugs have become an important strategy in innovative drug research. In addition, there are very few studies examining the efficacy of these novel drugs combining the effect of glucocorticoids and nitric oxide into one therapeutic option.

In this study, we evaluated and compared the anti-inflammatory activity of dexamethasone and nitric-oxide-releasing dexamethasone derivative NCX-1005. We hypothesized that NCX-1005 would decrease inflammation and oxidative imbalance and, thereby, alleviate the lung injury, which could result in improved lung function in an experimental model of ARDS in adult rabbits.

## 2. Materials and Methods

### 2.1. Nitrate Esters of Corticoid, Chemical Structure and Synthesis

**Figure 1:**
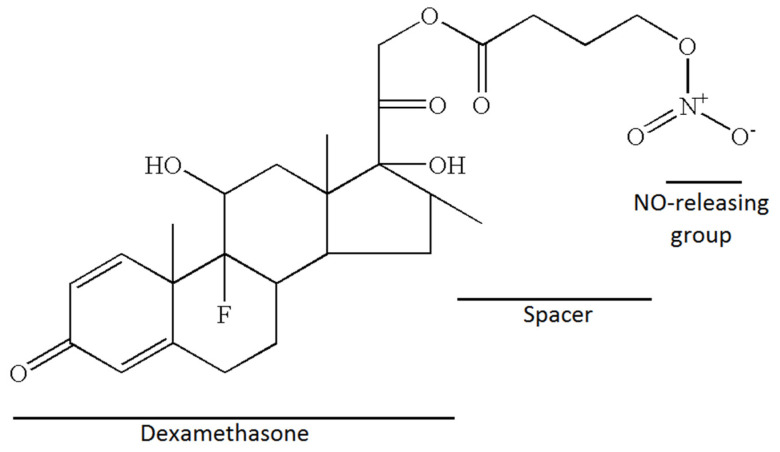
Chemical structure of NCX-1005: dexamethasone 21-(4-nitrooxybutyrate).

Nitric-oxide-releasing dexamethasone, NCX-1005, dexamethasone 21-(4-nitrooxybutyrate) was synthesized at NicOx Laboratories (Milan, Italy) (Figure 1). Position 21 was chosen because substitution of this position does not inhibit the biological activity of the native molecule. The solution of dexamethasone (3.5 g, 8.9 mmol) in tetrahydrofuran was treated with 4-bromobutyryl chloride (0.81 mL × 5) and potassium carbonate (0.98 g × 5) to obtain dexamethasone 21-(4-bromobutyrate) (1.5 g; m.p. 184–187 °C.; yield 31%). The mixture of dexamethasone 21-(4-bromobutyrate) and silver nitrate in acetonitrile was refluxed. After filtration and wash it yielded 1.27 g of pure dexamethasone 21-(4-nitrooxybutyrate) as a white solid (m.p. 183–185 °C.; yield 90%) (expired patent US7157450B2). The spacer between dexamethasone and the nitric oxide-releasing group assure the maintenance of the same chemical and pharmacological properties as those of dexamethasone phosphate [22].

### 2.2. Animal Instrumentation

Thirty-two adult New Zealand white rabbits, body weight (b.w.) 2.5 ± 0.2 kg were supplied by the certified animal breeding station (VELAZ, Praha, Czech Republic) and handled according to the Federation of European Laboratory Animal Science Association (FELASA) guidelines [23]. All animal experiments were approved by the National Veterinary Board of Slovakia and the local ethical committee of the Jessenius Faculty of Medicine in Martin, Comenius University.

The animals were instrumented as described previously [9]. After initial sedation, tracheotomy with endotracheal cannula insertion was performed, and the right femoral artery and vein were cannulated—the a. femoralis for blood sampling and arterial pressure monitoring and the vs. femoralis for continuous intravenous (*i.v.*) anaesthesia infusion (Zoletil, 10 mg/kg/h) and administration of therapy. The animals were mechanically ventilated (Aura V, Chirana, Slovakia) in volume-controlled mode with a tidal volume (V_T_) of 6 mL/kg, positive end-expiratory pressure (PEEP) of 5 cm H_2_O, respiratory rate (RR) of 40 breaths per minute (bpm), inspiratory to expiratory ratio (I:E) of 1:1, and inspired oxygen fraction (FiO_2_) of 1.0 for the entire duration of the experiment. Respiratory parameters and blood gases were recorded before (basal value, BV), after reaching ARDS, and 30′, 60′, 120′, 180′, and 240′ after the administration of therapy. Finally, the animals were euthanized under deep anaesthesia by the injection of potassium chloride *i.v.* All 24 animals survived the entire experimental procedure.

### 2.3. Experimental Protocol

After a 15 min stabilization period (V_T_ 6 mL/kg, PEEP 5 cm H_2_O, RR 40 bpm, I:E 1:1, FiO_2_ 1.0), the lung injury was induced by repetitive lung lavages with saline (30 mL/kg, 37 °C) via an endotracheal tube in the semi-upright right and left lateral positions of the animal followed by suction. The process was repeated with 2 min intervals of stabilization between the lavages until PaO_2_ in the arterial blood decreased to <26.7 kPa in two measurements at 5 and 15 min after the lavage, which equals moderate ARDS [1].

After the criteria of lung injury were fulfilled, the animals were randomized to three groups according to the therapy (n = 8 for each group): (1) ARDS group, without therapy; (2) DEX group, ARDS with 0.5 mg/kg dexamethasone *i.v.*, dexamethasone natrium phosphate (Dexamed sol inj, 8 mg/2 mL, Medopharm, Prague, Czech Republic); (3) NCX-1005 group, ARDS with 0.5 mg/kg NCX-1005 *i.v.*, dexamethasone 21-(4-nitrooxybutyrate) (NicOx Laboratories, Milan, Italy). The compound was dissolved in DMSO and diluted with saline as needed. The Control group represented healthy, ventilated, and non-treated animals (n = 8). After therapy or placebo, all animals were mechanically ventilated (V_T_ 6 mL/kg, PEEP 5 cm H_2_O, RR 40 bpm, I:E 1:1, FiO_2_ 1.0) for an additional 4 h.

### 2.4. Lung Function Parameters and Derived Indexes

Electrocardiographic monitoring, using subcutaneous electrodes, arterial pressure monitoring via a catheter in the a. femoralis connected to an electromanometer, and tracheal airflow and V_T_ measured by the heated Fleisch head connected to a pneumotachograph, was carried out continuously using multi-channel recorder PowerLab 8/30 (ADInstruments, Oxford, UK). Partial pressures of oxygen and carbon dioxide (PaO_2_, PaCO_2_), oxygen saturation (SaO_2_), and parameters of acid–base balance were measured by a blood gas analyser (RapidLab TM^348^, Bayer Diagnostics, Leverkusen, Germany). Ventilation parameters, V_T_, FiO_2_, minute ventilation, frequency, PIP, PEEP, mean airway pressure (MAP), static and dynamic lung compliance (C_stat_, C_dyn_), and airway resistance (Raw) were measured and calculated automatically by in-built sensors and software of the ventilator Aura V (Chirana, Slovakia). The following lung function parameters were calculated: P/F as the ratio between arterial PaO_2_ and FiO_2_; alveolar–arterial gradient (AaG) as: (FiO_2_ (Patm − PH_2_O) − PaCO_2_/0.8) − PaO_2_, where P_atm_ is the barometric pressure and PH_2_O is the pressure of water vapour; oxygenation index (OI) as (MAP × FiO_2_)/PaO_2_; and ventilation efficiency index (VEI) as 3800/((PIP − PEEP) × frequency × PaCO_2_).

### 2.5. White Blood Cells in the Blood and Bronchoalveolar Lavage Fluid

Samples of arterial blood for total leukocyte counting were taken at the beginning of the experiment (basal values, BV) and after 4 h of therapy (4 h Th). After sacrificing, lungs with trachea were excised and the left lung was lavaged with saline (three times, 10 mL/kg b.w.) to obtain bronchoalveolar lavage fluid (BALF). The total leukocyte count in blood and BALF was determined microscopically in a counting chamber after staining with Türk’s solution. The differential counts were estimated microscopically after staining with May–Grünwald/Giemsa–Romanowski stains. White blood cells, especially polymorphonuclear leukocytes, are the target cells for the quality evaluation of staining. The value of Romanowsky staining lies in its ability to produce a wide range of hues, allowing cellular components to be easily differentiated, i.e., nucleus—purple; lymphocyte plasma—blue; monocyte plasma—grey blue; neutrophil granule—light purple; eosinophil granule—red to dark purple; basophil granule—dark purple to black; thrombocytes—purple; erythrocytes—reddish.

### 2.6. Post-Mortem Analyses

Tissue samples from the right lung were either immediately shock frozen and stored at −70 °C until biochemical analyses were performed or used to assess the degree of lung oedema. Plasma samples of arterial blood were obtained by centrifugation (3000 rpm for 15 min, 4 °C). Levels of inflammatory and oxidation markers were determined in plasma and 10% (weight/volume) lung homogenate in 0.1 M phosphate buffer (PBS, pH 7.4). The concentration of IL-1β, TNFα, IL-6, and IL-8 was quantified using rabbit-specific ELISA kits (Cloud-Clone Corp., Houston, Texas, USA) and expressed in pg/mL. Oxidative modifications were determined using the following kits (Cell Biolabs Inc., San Diego, CA, USA): OxiSelect^TM^ Nitrotyrosine ELISA Kit for protein oxidation expressed in 3-nitrotyrosine nanomolar concentration (nM 3NT) and OxiSelect™ TBARS Assay Kit for lipid oxidation expressed as malondialdehyde in micromolar concentration (μM MDA). All biochemical analyses were performed in duplicates according to the manufacturers’ instructions.

The extent of the lung oedema was expressed as a wet-to-dry (W/D) lung weight ratio. Strips of the right lung were weighed before and after drying in an oven at 60 °C for 48 h to calculate the W/D ratio. The total protein content in the BALF was determined by the Bradford colourimetric method, as described previously [24].

### 2.7. Statistical Analysis

Statistical analysis was performed using GraphPad Prism 8.0.1 (USA). Data normality was tested using the Shapiro–Wilk test. All assessed variables were distributed normally; therefore, one-way ANOVA with Welch’s correction to test the groups and with Tukey post hoc test to test the parameters with dynamic changes was used. A *p* value below 0.05 was considered to be statistically significant. The results are presented as mean ± standard deviation (SD).

## 3. Results

### 3.1. Lung Function Parameters

**Figure 2:**
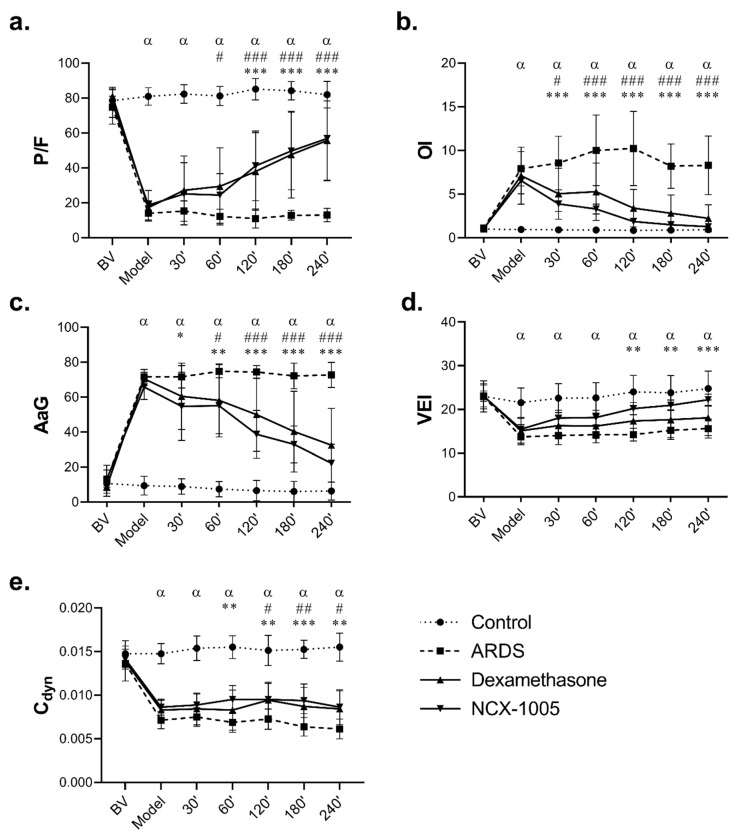
Changes in respiratory parameters. (**a**) The ratio of oxygen partial pressure to fraction of inspired oxygen (P/F, kPa), (**b**) oxygenation index, (**c**) alveolar–arterial gradient (AaG, kPa), (**d**) ventilation efficiency index (VEI), and (**e**) dynamic lung compliance (C_dyn_, mL/kPa) before (basal value, BV) and after ARDS induction and within 4 h after therapy administration in Control group, ARDS untreated group, and ARDS group treated with dexamethasone or with NCX-1005. Data are presented as mean ± SD. Statistical comparisons: for ARDS vs. Control ^α^ *p* < 0.001; for DEX vs. ARDS ^#^ *p* < 0.05, ^##^ *p* < 0.01, ^###^ *p* < 0.001; and for NCX-1005 vs. ARDS * *p* < 0.05, ** *p* < 0.01, *** *p* < 0.001.

Repetitive lung lavage caused a severe deterioration in all observed lung function parameters; the ratio of arterial oxygen partial pressure to fraction of inspired oxygen (P/F), oxygenation index (OI), alveolar–arterial gradient (AaG), ventilation efficiency index (VEI), dynamic compliance (C_dyn_), static compliance (C_stat_), mean airway pressure (MAP), and airway resistance (Raw) were significantly altered at timepoint “Model” in the time graphs (P/F ratio <26.7 kPa fulfilled the criteria for moderate ARDS according to the Berlin definition [1] in ARDS group compared to Control (all *p* < 0.001)). The deterioration of respiratory parameters persisted in the untreated ARDS group till the end of the experiment (Figure 2, Table 1). There were no significant differences across all animals in the baseline values (BVs) of the respiratory parameters, and no differences in the same parameters across both injured groups (ARDS vs. NCX-1005) at the timepoint Model (all *p* > 0.05).

Both therapies significantly improved lung function parameters (Figure 2, Table 1). At the first result-harvesting point after the administration of therapy (30′), NCX-1005 significantly improved OI, AaG, MAP, and Raw compared to the Control group (all *p* < 0.001), while the increase in P/F, VEI, C_stat_, and C_dyn_ occurred later, and these differences persisted till the end of the 4 h observation period, and with more potent effect compared to dexamethasone. Only NCX-1005 therapy significantly improved VEI, C_stat_, and PaCO_2_. At the last analysis after the administration of therapy (240′), *p* values for NCX-1005 compared to ARDS were as follows: P/F (<0.000; CI: 26.34, 61.42), OI (<0.000; CI: −10.05, −3.98), AaG (<0.000; CI: 34.66, 66.44), VEI (<0.000; CI: 2.18, 11.05), C_dyn_ (=0.004; CI: −0.004, −0.001), C_stat_ (=0.044; CI: −0.005, −0.000), MAP (<0.000; CI: 0.13, 0.40), Raw (<0.000; CI: 3.98, 12.92), SaO_2_ (<0.000; CI: −2.96, −9.34), and pH (=0.042; CI: −0.18, −0.00) (Figure 2, Table 1). There were no differences between the therapies, except for PaCO_2_ at 240′ NCX-1005 vs. DEX (*p* = 0.027; CI: 0.11, 2.66).

### 3.2. Total and Differential Leukocyte Count in Blood and Bronchoalveolar Lavage Fluid

Lavage-induced lung injury significantly shifted the total and differential leukocyte count in blood and bronchoalveolar lavage fluid (BALF) compared to that in the Control group (Table 2). In blood, both used therapies mitigated the shift in white blood cells compared to ARDS-untreated animals: for dexamethasone total count (*p* = 0.027; CI: −3.47, −0.23), neutrophils (*p* = 0.003; CI: −47.41, −14.70), lymphocytes (*p* = 0.003; CI: 14.25, 45.43); and for NCX-1005 total count (*p* = 0.019; CI: −3.59, −0.36), neutrophils (*p* = 0.029; CI: −41.11, −7.52), lymphocytes (*p* = 0.037; CI: 7.41, 39.49) compared to the ARDS group. In BALF, both therapies inhibited cell infiltration into the lungs—total count for DEX (*p* = 0.000, CI: 43.65, 73.40) and for NCX-1005 (*p* = 0.000, CI: 42.36, 72.12). When comparing the differential leukocyte count in BALF, only NCX-1005 therapy significantly increased monocyte count (*p* = 0.007; CI: −62.93, −7.94) and decreased neutrophils (*p* = 0.025; CI: 9.78, 61.04) compared to the ARDS group. We observed significant differences between the therapies, DEX vs. NCX-1005 for monocytes (*p* = 0.025; CI: −41.42, −3.10) and neutrophils (*p* = 0.021; CI: 3.68, 40.79) (Table 2).

### 3.3. Inflammation and Oxidation in Plasma and Lung Tissue

**Figure 3:**
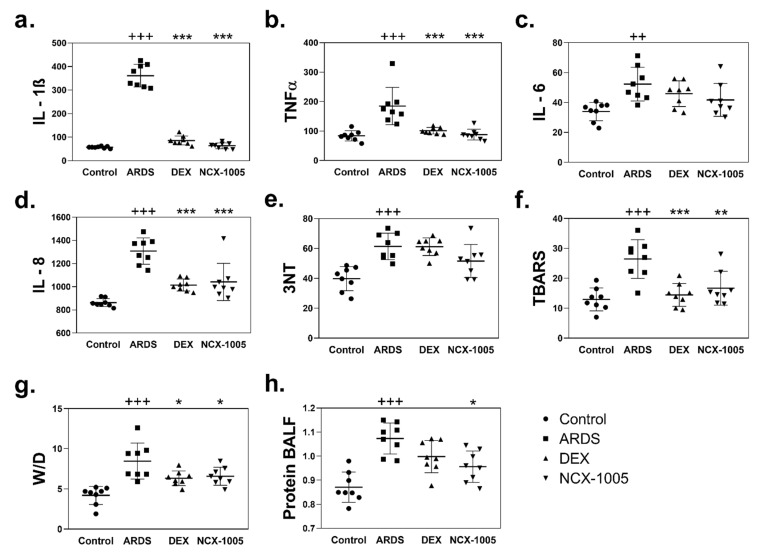
Degree of inflammation, oxidation, and lung oedema formation in lung. Levels of cytokines (**a**) IL-1β, (**b**) TNFα, (**c**) IL-6, and (**d**) IL-8 (all in pg/mL); oxidation of (**e**) protein (3-nitrotyrosine, 3NT, nmol) and (**f**) lipid (thiobarbituric acid-reactive substances TBARS, mmol MDA) in lung homogenates; (**g**) lung oedema expressed as wet–dry (W/D) lung weight ratio; and (**h**) protein content in bronchoalveolar lavage fluid (BALF, μg/mL) in Control group, ARDS-untreated group, and ARDS group treated with dexamethasone or NCX-1005. Data are presented as individual values with mean ± SD. Statistical comparisons: for ARDS vs. Control ^++^ *p*<0.01, ^+++^*p* < 0.001 and for DEX and NCX-1005 vs. ARDS * *p* < 0.05, ** *p* < 0.01, *** *p* < 0.001.

Levels of inflammatory cytokines, tumour necrosis factor-alpha (TNFα) and interleukin (IL)-1β, -6, -8, and markers of oxidation, 3-nitrotyrosine (3NT) and thiobarbituric acid-reactive substances (TBARS) were significantly elevated in the lung tissue and plasma after the repetitive lung lavage in the ARDS group compared to that in the Control (Figure 3, Table 3). The effect of both therapies was reflected in decreased levels of observed biochemical markers. In plasma (Table 3), only NCX-1005 significantly reduced the level of IL-8 compared to that in ARDS group (*p* = 0.025; CI: 54.21, 763.20). In lung tissue (Figure 3a,b,d,f), decreased levels of cytokines and TBARS were observed after both therapies compared to that in the ARDS group simultaneously, for DEX: IL-1β (*p* = 0.000; CI: 234.10, 311.30), TNFα (*p* = 0.000; CI: 29.79, 117.30), IL-8 (*p* = 0.000; CI: 157.70, 432.80), and TBARS (*p* = 0.001; CI: 5.10, 18.95); and for NCX-1005: IL-1β (*p* = 0.000; CI: 257.60, 334.90), TNFα (*p* = 0.000; CI: 41.32, 132.20), IL-8 (*p* = 0.000; CI: 129.50, 404.60), TBARS (*p* = 0.005; CI: 2.66, 16.51), and 3NT was borderline (*p* = 0.058; CI: −0.38, 20.40). There were no statistically significant differences between the therapies DEX vs. NCX-1005.

### 3.4. Lung Oedema and Protein Content in Bronchoalveolar Lavage Fluid

Lung oedema was expressed as a wet–dry lung weight ratio (W/D) of lung tissue increased after the lavage-induced lung injury compared to controls (*p* = 0.000; CI: 2.34, 6.23) and, similarly, total protein content in bronchoalveolar lavage fluid (*p* = 0.000; CI: 0.12, 0.29) for ARDS vs. Control. Both therapies significantly reduced the lung oedema formation compared to that in the ARDS group, for dexamethasone (*p* = 0.020; CI: 0.29, 3.94) and NCX-1005 (*p* = 0.021; CI: 0.31, 3.45). Total protein content was significantly reduced only after NCX-1005 therapy (*p* = 0.019; CI: 0.02, 0.20) compared to the untreated ARDS group (Figure 3g,h).

## 4. Discussion

ARDS represents stereotypic progress through several phases after pulmonary or extrapulmonary insults. In the initial phase, alveolar macrophages secrete mediators that lead to the accumulation of inflammatory cells in the lung. Activated alveolar epithelial cells and neutrophils in alveolar spaces can induce an overwhelming inflammation leading to lung tissue injury. Migration of inflammatory cells across the vascular endothelial and alveolar epithelial surfaces and release of pro-inflammatory mediators could lead to pathologic vascular permeability, gaps in the alveolar epithelial barrier, and necrosis of type I and II alveolar cells. Intravascular coagulation in the alveolar capillaries leads to the production of microthrombi. The result of these changes is pulmonary oedema, surfactant inactivation, and the deposition of dead cells and debris along the alveoli (hyaline membranes), which decrease pulmonary compliance and deteriorate gas exchange in the lungs [3,25]. The inflammation induced by immune cells is modulated by the inflammatory mediators including IL-1β, IL-6, TNF-α, and IL-18 [26]. Based on the aforementioned facts, anti-inflammatory therapy appears to be the first choice of ARDS, and therapy with glucocorticoids may be of benefit. GCs could suppress inflammation and oxidation, promote alveolar fluid clearance, stimulate surfactant production, and reduce neutrophil recruitment [27]. Our study is focused on the effects of intravenously administered dexamethasone (DEX) and nitro-dexamethasone (NCX-1005) to mitigate the inflammatory response, decrease pro-inflammatory cytokine production, oxidative damage, and oedema formation, which finally could lead to improvement in respiration and gas exchange during an acute phase of experimental ARDS.

Damage of the alveolar–capillary membrane caused by inflammation leads to an influx of fluid and inflammatory activated cells into the alveoli. This process could negatively affect surfactant function and, thus, respiration. Repeated lung lavage led to the deterioration of the lung function parameters (P/F, OI, AaG, VEI, C_dyn_, MAP, C_stat_, Raw, PaCO_2_) within minutes after the insult, which is consistent with the findings of other authors [28,29,30]. Respiratory failure in animals with ARDS persisted until the end of the experiment probably due to surfactant dysfunction, which could be caused by interaction with leaked plasma proteins (albumin and fibrinogen) [31]. Corticosteroids have been demonstrated to modulate pulmonary vasomotor activity in hypertensive lung injuries by the influence of their release and action in the lungs [32,33]. The tendency to improve gas exchange after glucocorticoid administration, documented in the present study, may be due to its permissive effects on local hypoxic vasoconstriction, resulting in the redistribution of the pulmonary blood flow to the better-ventilated areas [33,34]. Reduced endothelial damage and vascular permeability, accompanied by a decrease in pulmonary microvascular pressures, may also play a role in pulmonary oedema generation [32,33,35]. In our study, the administration of dexamethasone (DEX) and NCX-1005 improved lung function parameters comparably. DEX and NCX-1005 therapies enhanced gas exchange compared to animals with ARDS. After DEX therapy, we observed a rapid improvement in the oxygenation index and the alveolar–arterial gradient within the first 30 min after administration of the therapy, and this beneficial effect persisted till the end of the experiment. A similar improvement with the same persistent effect was observed after NCX-1005 administration. There were no significant differences in lung function parameters between these two therapies. Previous studies using GCs in the early phase of ARDS confirm our results. Several experimental [8,9,36] and clinical studies [37,38,39,40,41] showed that systemically administered GCs (e.g., methylprednisolone, dexamethasone, prednisolone) could improve lung function due to effective improvement in oxygenation and ventilation parameters and, thus, contribute to the reduction of the oxygen-dependency period in ICU and hospital stay.

In ARDS, it is essential to manage the systemic and also pulmonary inflammatory response. The early phase of ARDS is pathophysiologically characterized by a neutrophil-mediated inflammation, and lung cell injury and apoptosis, with the subsequent influx of protein-rich fluid into the alveoli and oedema formation. Therapy using glucocorticoids (GCs) may be beneficial due to their actions. The anti-inflammatory action of GCs has been widely demonstrated in several studies using animal models of inflammation [8,9,42,43,44]. In our experimental model, the repeated lung lavages could contribute to diffuse alveolar injury, which is associated with a massive influx of leukocytes from the circulation into the lung and alveolar spaces. We observed a significant increase in the total count of cells with dominant neutrophils in bronchoalveolar lavage fluid (BALF) in the ARDS group compared Control group. Infiltration of neutrophils in the inflamed lung is a hallmark of ARDS [45]. Activated neutrophils trigger oxidative damage and release proteases leading to lung damage. The induction of epithelial and endothelial injury contributes to the development of alveolar oedema and hypoxaemia, as well as exacerbating the pro-inflammatory state. It should be recognized that neutrophil migration into the lung without concomitant activation does not induce tissue injury [46]. Neutrophil activation is initiated in response to inflammation. The process starts with the recruitment of neutrophils to the inflamed site from blood circulation via their adhesion and migration, under the guidance of chemoattractant gradients [47,48,49]. Once neutrophils enter the interstitial space, they use the gradient of chemoattractants, such as IL-8, which results in the oxidative burst. Dexamethasone therapy slightly decreased the percentage of neutrophils; however, NCX-1005 therapy significantly affected the percentage of monocytes and neutrophils in BALF compared to the ARDS group and also compared to DEX group. A similar result was observed in the previous study when the nitro-derivative was more potent than the parent compound in reducing the accumulation of neutrophils [50]. According to the previous studies, administration of the higher dose of dexamethasone (1.0 instead of 0.5 mg/kg) significantly affected inflammatory cell count in BALF [8,9].

An influx of neutrophils to the lung and their activation and burst leads to inflammation, which plays a key role in the progression of ARDS. In our experimental condition, we observed an increased level of pro-inflammatory cytokines (e.g., TNFα, IL-1β, IL-6, and IL-8) in the plasma and lung homogenates. These results are consistent with previous findings [51,52,53]. The anti-inflammatory actions of GCs by inhibiting cytokine production are particularly relevant [54]. GCs block the transcription of NF-B-dependent pro-inflammatory genes, such as IL-1, IL-3, IL-4, IL-5, IL-6, IL-8, TNF, and granulocyte-macrophage colony-stimulating factor. GCs also reduce fibrogenesis via acting on the IL-1 receptor antagonist as well as the anti-inflammatory cytokines IL-4, IL-10, and IL-13. Genes that encode pro-inflammatory cytokines are turned off by GCs, while genes that encode anti-inflammatory cytokines are turned on [55]. In our experiments, the administration of dexamethasone and NCX-1005 decreased levels of observed pro-inflammatory cytokines in the lung and plasma compared to that in the ARDS group. We did not observe differences among the treated groups. Only NCX-1005 therapy decreased the level of IL-8 in plasma. It has been reported previously, that low doses of GCs prevent an extended cytokine response and might accelerate the resolution of pulmonary and systemic inflammation in pneumonia [56].

Overactivation of neutrophils, especially neutrophil oxidative burst, can cause tissue injury by the oxidation of proteins and lipids. Neutrophils release cytotoxic and immune-cell-activating agents such as proteinases, cationic polypeptides, cytokines, and free radicals of reactive oxygen and nitrogen species (RONS) [57]. In our experiments, after lavage-induced lung injury, protein nitrosylation (3-nitrotyrosine, 3NT) and lipid peroxidation (TBARS) significantly increased in the lung and plasma. Neutrophil-induced damage of proteins and lipids through their oxidation was confirmed in several studies, with results demonstrating increased levels of RONS in the alveolar spaces during ARDS [58,59,60]. In the lung homogenates, TBARS was significantly decreased after therapy with DEX and NCX-1005 compared to that in untreated ARDS.

The actions of inflammatory mediators and bioactive compounds such as RONS can damage endothelial and epithelial cells, leading to increase permeability across the alveolar–capillary membrane and pulmonary oedema formation. Large numbers of activated neutrophils can damage the alveolar epithelium, probably by the release of toxic intracellular molecules that induce dissolution of tight junctions as well as apoptosis and necrosis of alveolar epithelial type I and type II cells [57]. Extensive alveolar epithelial injury results in the formation of alveolar oedema containing high-molecular-weight serum proteins, worsening gas exchange and increasing the risk of disordered repair [61,62]. In our study, the degree of lung oedema was calculated from a ratio of wet and dry lung weight (wet/dry, W/D). The ARDS group had a significantly higher W/D value compared to controls, indicating increased pulmonary fluid accumulation in the pulmonary interstitium. Hand in hand, a significantly higher level of total proteins level in BALF was observed in the ARDS group. Similar findings were reported previously [29]. Both therapies comparatively decreased lung oedema formation. The anti-oedematous effect of NCX-1005 was visible also in significantly decreasing the protein content in BALF compared to that the ARDS group.

There are some potential limitations of our research. To begin with, no single animal model can accurately mimic all of the symptoms of ARDS in people. One of the most extensively used ARDS animal models is alveolar lavage with warmed normal saline, which was used in this investigation. However, this is primarily a surfactant depletion model, which causes lung injury similar to human ARDS in terms of oxygenation, pulmonary compliance and atelectasis, and oedema but induces less macrophage and neutrophil infiltration unless another injury is added, such as mechanical ventilation [63]. Furthermore, because surfactant suppresses neutrophil respiratory activation and has an antioxidant effect on alveolar macrophages, it can interfere with the lung inflammatory/immune response and oxidative metabolism [24]. The nitro-steroids have been found to offer an enhanced anti-inflammatory and anti-arthritic effect in animal models at the same level as the parent compound, but they prevent hypertension induced by GCs [50].

In conclusions, the nitric-oxide-releasing dexamethasone derivative NCX-1005 has the potential to be an effective drug with an anti-inflammatory effect. NCX-1005 prevented the migration of neutrophils into the lungs and modulated their activation, which lead to decreased levels of cytokines and oxidative modifications in lung tissue and reduction in lung oedema formation. Inhibition of local inflammation could alleviate respiratory failure showed in the improvement of the observed lung function parameters. In addition, the activities of NCX-1005 are comparable to those of dexamethasone during the early phase of ARDS, the effect of which is known [9,63]. It is difficult to argue about which part of the composite molecule contributed more significantly to the overall effect. However, NCX-1005 can be of benefit in the therapy of ARDS, but further research in this field is necessary.

## Figures and Tables

**Table 1:** Respiratory parameters. Mean airway pressure (MAP), static lung compliance (C_stat_), airway resistance (Raw), partial pressure of carbon dioxide (PaCO_2_), oxygen saturation (SaO_2_), and pH before (basal value, BV) and after ARDS induction and within 4 h after therapy administration in Control group, ARDS-untreated group, and ARDS group treated dexamethasone or with NCX-1005.

	BV	Model	30′	60′	120′	180′	240′
MAP (kPa)							
Control	0.78 ± 0.04	0.76 ± 0.05	0.73 ± 0.05	0.72 ± 0.04	0.71 ± 0.03	0.72 ± 0.04	0.73 ± 0.05
ARDS	0.81 ± 0.07	1.13 ± 0.09 ^†††^	1.08 ± 0.07 ^†††^	1.09 ± 0.08 ^†††^	1.15 ± 0.11 ^†††^	1.16 ± 0.11 ^†††^	1.16 ± 0.12 ^†††^
DEX	0.81 ± 0.04	1.06 ± 0.11	0.94 ± 0.10 ^#^	1.00 ± 0.08	0.91 ± 0.09 ^###^	0.94 ± 0.16 ^###^	0.97 ± 0.19 ^###^
NCX-1005	0.79 ± 0.04	1.02 ± 0.11	0.93 ± 0.08 *	0.93 ± 0.10 *	0.91 ± 0.15 ***	0.89 ± 0.19 ***	0.90 ± 0.18 ***
C_stat_ (mL/kPa)							
Control	0.016 ± 0.001	0.016 ± 0.001	0.017 ± 0.001	0.017 ± 0.002	0.017 ± 0.002	0.017 ± 0.001	0.017 ± 0.001
ARDS	0.015 ± 0.003	0.009 ± 0.002 ^†††^	0.009 ± 0.001 ^†††^	0.008 ± 0.002 ^†††^	0.008 ± 0.001 ^†††^	0.008 ± 0.001 ^†††^	0.008 ± 0.001 ^†††^
DEX	0.016 ± 0.002	0.009 ± 0.002	0.010 ± 0.003	0.010 ± 0.003	0.011 ± 0.002	0.010 ± 0.003	0.010 ± 0.002
NCX-1005	0.016 ± 0.002	0.009 ± 0.001	0.010 ± 0.001	0.011 ± 0.001 *	0.011 ± 0.001 *	0.011 ± 0.001 *	0.010 ± 0.001 *
Raw (kPa/l/s)							
Control	4.68 ± 0.76	4.79 ± 0.95	5.27 ± 0.90	4.81 ± 0.81	4.99 ± 0.72	4.67 ± 0.67	4.58 ± 0.82
ARDS	5.02 ± 1.51	12.17 ± 5.35 ^†††^	14.44 ± 5.27 ^†††^	15.96 ± 5.76 ^†††^	17.02 ± 6.95 ^†††^	16.19 ± 7.14 ^†††^	17.14 ± 6.67 ^†††^
DEX	4.72 ± 0.77	7.69 ± 1.72	8.70 ± 2.23 ^##^	8.99 ± 1.81 ^###^	7.66 ± 1.90 ^###^	8.58 ± 3.36 ^###^	9.07 ± 2.88 ^###^
NCX-1005	4.28 ± 1.01	9.12 ± 1.54	7.88 ± 2.08 **	7.77 ± 2.69 ***	8.01 ± 4.10 ***	8.10 ± 3.43 ***	8.69 ± 3.47 ***
PaCO_2_ (kPa)							
Control	4.23 ± 0.69	4.49 ± 0.64	4.29 ± 0.61	4.29 ± 0.71	4.06 ± 0.78	4.08 ± 0.68	3.93 ± 0.70
ARDS	4.05 ± 0.61	7.03 ± 1.00 ^†††^	6.82 ± 1.33 ^†††^	6.76 ± 1.05 ^†††^	6.75 ± 0.75 ^†††^	6.37 ± 0.69 ^†††^	6.41 ± 0.88 ^†††^
DEX	3.99 ± 0.21	6.5 ± 1.01	5.97 ± 0.86	6.17 ± 0.97	5.86 ± 0.99	5.94 ± 1.53	5.70 ± 1.31
NCX-1005	4.10 ± 0.63	6.06 ± 1.12	5.77 ± 1.33	5.61 ± 1.28	4.91 ± 0.95 ***	4.67 ± 0.73 **	4.32 ± 0.72 ***^@^
SaO_2_ (%)							
Control	99.90 ± 0.00	99.90 ± 0.00	99.90 ± 0.00	99.90 ± 0.00	99.90 ± 0.00	99.90 ± 0.00	99.89 ± 0.04
ARDS	99.90 ± 0.00	94.34 ± 3.59	95.83 ± 2.06	89.47 ± 12.42 ^†††^	88.74 ± 9.07 ^†††^	91.24 ± 5.19 ^††^	84.67 ± 14.41 ^†††^
DEX	99.90 ± 0.00	97.39 ± 1.72	97.47 ± 1.92	97.16 ± 2.48 ^##^	98.76 ± 1.10 ^###^	99.20 ± 0.79 ^##^	99.49 ± 0.49 ^###^
NCX-1005	99.91 ± 0.04	96.63 ± 4.49	96.41 ± 5.88	99.00 ± 1.20 ***	99.69 ± 0.24 ***	99.76 ± 0.11 **	99.83 ± 0.09 ***
pH							
Control	7.49 ± 0.05	7.44 ± 0.08	7.40 ± 0.03	7.37 ± 0.03	7.33 ± 0.07	7.29 ± 0.04	7.27 ± 0.04
ARDS	7.54 ± 0.03	7.23 ± 0.04 ^†††^	7.23 ± 0.06 ^†††^	7.21 ± 0.06 ^†††^	7.15 ± 0.06 ^†††^	7.12 ± 0.07 ^†††^	7.08 ± 0.04 ^†††^
DEX	7.47 ± 0.08	7.25 ± 0.05	7.26 ± 0.04	7.24 ± 0.05	7.23 ± 0.04	7.19 ± 0.07	7.15 ± 0.07
NCX-1005	7.48 ± 0.09	7.26 ± 0.09	7.27 ± 0.11	7.25 ± 0.10	7.23 ± 0.09	7.19 ± 0.10	7.17 ± 0.09 *

Data are presented as mean ± SD. Statistical comparisons: for ARDS vs. Control ^††^ *p* < 0.01, ^†††^ *p* < 0.001; for DEX vs. ARDS ^#^ *p* < 0.05, ^##^ *p* < 0.01, ^###^ *p* < 0.001; for NCX-1005 vs. ARDS * *p* < 0.05, ** *p* < 0.01, *** *p* < 0.001; and for DEX vs. NCX-1005 ^@^ *p* < 0.05.

**Table 2:** White blood cell count. Total and differential leukocyte count in the blood before (basal value, BV) and in the 4 h of the therapy (Th); and in the bronchoalveolar lavage fluid (BALF) in Control group, ARDS-untreated group, and ARDS group treated with dexamethasone or with NCX-1005.

**BLOOD**					
		**Control**	**ARDS**	**DEX**	**NCX-1005**
Total leukocyte count (×10^6^/mL)					
	BV	2.56 ± 1.72	2.54 ± 2.27	2.57 ± 1.71	2.65 ± 1.59
	4 h Th	3.11 ± 2.13	1.15 ± 0.61 ^†^	3.00 ± 0.91 *	3.13 ± 1.60 *
Differential count (%)					
Neutrophils	BV	2.54 ± 1.82	2.07 ± 1.58	2.04 ± 1.20	2.19 ± 2.55
	4 h Th	56.23 ± 13.68	19.59 ± 5.50 ^††^	50.64 ± 17.67 **	43.90 ± 10.71 *
Lymphocytes	BV	94.18 ± 3.95	96.23 ± 2.51	95.47 ± 2.06	95.16 ± 3.68
	4 h Th	42.10 ± 13.26	77.43 ± 7.69 ^††^	47.59 ± 17.34 **	53.98 ± 10.56 *
Monocytes	BV	1.13 ± 0.55	0.93 ± 0.34	1.21 ± 0.52	0.86 ± 0.29
	4 h Th	1.32 ± 0.44	0.78 ± 0.39	1.07 ± 0.60	1.01 ± 0.62
Eosinophils	BV	0.85 ± 0.71	0.96 ± 1.04	1.27 ± 1.63	1.49 ± 1.43
	4 h Th	0.36 ± 0.31	0.44 ± 0.42	0.70 ± 0.55	0.94 ± 0.54
**BALF**					
		**Control**	**ARDS**	**DEX**	**NCX-1005**
Total count (×10^3^/mL)		2.29 ± 0.76	66.67 ± 20.41 ^†††^	8.14 ± 6.34 ***	9.43 ± 7.00 ***
Monocytes (%)		96.22 ± 3.18	20.43 ± 11.44 ^†††^	33.60 ± 28.47	55.86 ± 17.49 **^@^
Neutrophils (%)		2.74 ± 2.31	76.67 ± 12.08 ^†††^	63.50 ± 27.34	41.26 ± 18.26 *^@^
Eosinophils (%)		0.85 ± 0.76	3.23 ± 1.87 ^†^	2.74 ± 1.69	3.19 ± 1.79

Data are presented as means ± SD. Statistical comparisons: for ARDS vs. Control ^†^ *p* < 0.05, ^††^ *p* < 0.01, ^†††^ *p* < 0.001; for DEX and NCX-1005 vs. ARDS * *p* < 0.05, ** *p* < 0.01, *** *p* < 0.001; and for DEX vs. NCX-1005 ^@^ *p* < 0.05.

**Table 3:** Degree of inflammation and oxidation in plasma at the end of experiment. Levels of cytokines, IL-1β, TNFα, IL-6, and IL-8 (all in pg/mL) and protein (3-nitrotyrosine, 3NT, nmol) and lipid oxidation (thiobarbituric acid-reactive substances TBARS, mmol MDA) in Control group, ARDS-untreated group, and ARDS group treated with dexamethasone or with NCX-1005. Data are presented as means ± SD. Statistical comparisons: for ARDS vs. Control ^†^ *p* < 0.05, ^††^ *p* < 0.01, ^†††^ *p* < 0.001; and for NCX-1005 vs. ARDS * *p* < 0.05.

	3NT	TBARS	IL-1β	TNFα	IL-6	IL-8
Control	18.1 ± 0.9	6.4 ± 2.4	14.5 ± 16.7	57.5 ± 60.4	9.7 ± 6.3	424.0 ± 270.9
ARDS	21.9 ± 4.1 ^†^	10.0 ± 2.6 ^††^	130.8 ± 98.2 ^†^	251.6 ± 117.7 ^††^	88.0 ± 66.9 ^†^	1050.0 ± 308.7 ^†††^
DEX	18.3 ± 1.3	7.2 ± 0.7	81.5 ± 63.1	115.2 ± 145.8	58.4 ± 62.1	677.2 ± 449.1
NCX-1005	19.5 ± 2.4	7.9 ± 1.1	81.3 ± 83.1	151.4 ± 80.8	66.1 ± 46.5	641.7 ± 289.1 *

## Data Availability

The data presented in this study are available on request from corresponding author. These are raw data, which are published in this study in the form of an average and standard deviation. The data are not publicly available due to preliminary study and continuing research.
